# EAK proteins: novel conserved regulators of *C. elegans* lifespan

**DOI:** 10.18632/aging.100214

**Published:** 2010-10-25

**Authors:** Travis W. Williams, Kathleen J. Dumas, Patrick J. Hu

**Affiliations:** ^1^ Life Sciences Institute, University of Michigan, Ann Arbor, MI 48109, USA; ^2^ Cellular and Molecular Biology Graduate Program, University of Michigan Medical School, Ann Arbor, MI 48109, USA; ^3^ Department of Internal Medicine, University of Michigan Medical School, Ann Arbor, MI 48109, USA; ^4^ Department of Cell and Developmental Biology, University of Michigan Medical School, Ann Arbor, MI 48109, USA

**Keywords:** C. elegans, aging, lifespan, longevity, insulin signaling, Akt, FoxO, eak, diabetes, cancer, osteoporosis

## Abstract

FoxO transcription factors (TFs) extend lifespan in invertebrates and may participate in the control of human longevity. The role of FoxO TFs in lifespan regulation has been studied most extensively in *C. elegans*, where a conserved insulin/insulin-like growth factor signaling (IIS) pathway and the germline both control lifespan by regulating the subcellular localization of the FoxO transcription factor DAF-16. Although the control of FoxO activity through modulation of its subcellular localization is well established, nuclear translocation of FoxO is not sufficient for full FoxO activation, suggesting that undiscovered inputs regulate FoxO activity after its translocation to the nucleus. We have recently discovered a new conserved pathway, the EAK (enhancer-of-*akt*-1) pathway, which acts in parallel to the Akt/PKB family of serine-threonine kinases to regulate DAF-16/FoxO activity. Whereas mutation of Akt/PKB promotes the nuclear accumulation of DAF-16/FoxO, mutation of *eak* genes increases nuclear DAF-16/FoxO activity without influencing DAF-16/FoxO subcellular localization. Thus, EAK proteins regulate the activity of nuclear DAF-16/FoxO. Two EAK proteins, EAK-2/HSD-1 and EAK-7, influence *C. elegans* lifespan and are conserved in mammals. The discovery of the EAK pathway defines a new conserved FoxO regulatory input and may have implications relevant to aging and the pathogenesis of aging-associated diseases.

## FoxO TFs in longevity and aging-associated disease

The role of FoxO TFs in lifespan control was first established in *C. elegans*, where reduction of IIS or ablation of the germline extends lifespan in a DAF-16/FoxO-dependent manner [1,2]. FoxO also promotes lifespan extension in *Drosophila* [3,4]. Although reduction of IIS also promotes longevity in mammals [5,6,7], whether or not this requires FoxO TFs remains to be determined. Multiple studies indicate that specific FoxO polymorphisms are associated with longevity in cohorts of extremely long-lived humans [8,9,10,11,12,13], suggesting that FoxO TFs may also regulate lifespan in humans.

While a direct connection between mammalian FoxO TFs and lifespan control has not yet been established experimentally, recent work in conditional knockout mice supports a role for FoxO TFs in modulating phenotypes that are reminiscent of aging-associated diseases in humans. For example, mice lacking FoxO1, FoxO3, and FoxO4 develop thymic lymphomas and hemangiomas, indicating that FoxO TFs are bonafide tumor suppressors [14]. Furthermore, deletion of FoxO TFs in osteoblasts results in reduced bone mass secondary to increased osteoblast apoptosis [15,16], suggesting that FoxO TFs are protective against osteoporosis. In contrast to these apparent salubrious effects of FoxO TFs, FoxO1 can contribute to metabolic dysregulation similar to that observed in Type 2 diabetes, as FoxO1 haploinsufficiency protects mice against insulin resistance induced by a high-fat diet [17], and both liver-specific and osteoblast-specific FoxO1 deletion ameliorate glucose intolerance in mouse models of insulin resistance [18,19,20]. Thus, in mammals, FoxO TFs have context-dependent effects on the development of phenotypes associated with age-related disease. Elucidating the regulatory mechanisms that maintain the balance of FoxO TF activity may prove to be crucial for understanding and combating the progression of age-related disease.

## DAF-16/FoxO regulation in the control of *C. elegans* lifespan

DAF-16/FoxO is required for *C. elegans* lifespan modulation by IIS and the germline [1,2], as well as in some contexts of dietary restriction [21]. Reduction of IIS and ablation of the germline both extend lifespan by increasing DAF-16/FoxO activity. Neither intervention increases lifespan in *daf-16/FoxO* null mutants [1,2], indicating that DAF-16/FoxO is the critical target of IIS and the germline in lifespan control.

IIS and the germline both inhibit DAF-16/FoxO by promoting its cytoplasmic sequestration. When IIS is reduced, or when the germline is ablated, DAF-16/FoxO translocates to the nucleus [22,23,24], where it executes gene regulatory programs that promote longevity. IIS induces the phosphorylation of DAF-16/FoxO by Akt/Protein Kinase B (PKB) [23,24], which results in the cytoplasmic sequestration of DAF-16/FoxO through its direct association with 14-3-3 pro- teins [25,26]. The molecular requirements for DAF-16/FoxO nuclear translocation in the context of reduced IIS have not been fully delineated but may include serine-threonine kinases such as JNK-1 [27] and CST-1 [28].

How the germline promotes the cytoplasmic sequestration of DAF-16/FoxO is not entirely understood. Notably, germline ablation extends lifespan in animals with reduced IIS [2]. In animals lacking a germ-line, translocation of cytoplasmic DAF-16/FoxO to the nucleus requires the nuclear receptor DAF-12 [29] and its steroid hormone ligands [29,30], which are known as dafachronic acids (DAs) [31]. The conserved protein KRI-1 is required for DAF-16/FoxO nuclear translocation in animals lacking a germline but largely dispensable for DAF-16/FoxO nuclear localization in animals with reduced IIS [29]. In aggregate, these observations suggest that IIS and the germline control the subcellular localization of DAF-16/FoxO via distinct mechanisms.

## Nuclear translocation is not sufficient for full DAF-16/FoxO activation

Although nuclear localization of DAF-16/FoxO is clearly necessary for DAF-16/FoxO-dependent lifespan extension, multiple lines of evidence indicate that it is not sufficient for full DAF-16/FoxO activation. For example, a DAF-16/FoxO mutant lacking all four canonical Akt/PKB phosphorylation sites localizes to the nucleus but fails to fully extend lifespan [24,29]. This indicates that a second pathway acts in parallel to Akt/PKB and the germline to inhibit the activity of nuclear DAF-16/FoxO.

## A genetic screen for molecules that regulate nuclear DAF-16/FoxO activity

To identify components of this parallel DAF-16/FoxO regulatory pathway, we exploited the fact that in larvae, DAF-16/FoxO promotes developmental arrest in an alternative larval stage called dauer that is morphologically distinct from reproductively developing larvae [32]. We performed a genetic screen for mutants that enhance the weak dauer-constitutive phenotype of an *akt-1* null mutant (*eak* screen). We identified 21 independent mutants that define seven *eak* genes, six of which have been cloned (Table 1) [33,34,35]. Strikingly, five of the six cloned *eak* genes are expressed specifically in the two endocrine XXX cells [34,35,36]; in contrast, *eak-7* is expressed in the XXX cells as well as multiple other tissues [33]. The phenotypic similarity of all *eak* single mutants and the observation that no *eak;eak* double mutant combination tested to date exhibits phenotypic enhancement compared to *eak* single mutants indicate that the EAK proteins are components of a single pathway.

**Table 1.** Seven *eak* genes. The molecular identity of *eak-1* is not known.

Gene	Predicted product	Human ortholog	Expression pattern
*eak-1*	?	?	?
*eak-2*	*hsd-1*	Sdr42e1	XXX cells
*eak-3*	N-myristoylated protein	?	XXX cells
*eak-4*	N-myristoylated protein	?	XXX cells
*eak-5*	*sdf-9*	?	XXX cells
*eak-6*	Protein tyrosine phosphatase	?	XXX cells^1^
*eak-7*	N-myristoylated TLDc protein	KIAA1609	XXX cells, neurons, intestine, other tissues

1 *eak-6* is also expressed in the M1 pharyngeal motor neuron

EAK proteins and AKT-1 both control DAF-16/FoxO target gene expression. However, in contrast to AKT-1, which inhibits DAF-16/FoxO activity by promoting its translocation from the nucleus to the cytoplasm, EAK proteins inhibit nuclear DAF-16/FoxO activity without influencing its subcellular localization [33,34,35]. Collectively, the data indicate that EAK proteins define a new conserved endocrine pathway that acts in parallel to Akt/PKB to regulate the activity of nuclear DAF-16/FoxO. Two *eak* genes, *eak-2/hsd-1* and *eak-7,* have putative mammalian orthologs based on comparative sequence analysis [33,34].

## EAK-2/HSD-1

EAK-2 is allelic to the 3β-hydroxysteroid dehydro-genase family member HSD-1 [34,37]. HSD-1 is thought to participate with the cytochrome P450 DAF-9 in the biosynthesis of Δ^4^-dafachronic acid (Δ^4^-DA) [31,37]; the related steroid Δ^7^-DA is synthesized by the Rieske oxygenase family member DAF-36 and DAF-9 [31,38]. Both Δ^4^- and Δ^7^-DA are high-affinity ligands for the nuclear receptor DAF-12 [31].

The identification of *hsd-1* mutations in the *eak* screen implicates steroid hormone signaling in the regulation of nuclear DAF-16/FoxO activity. In support of this, we have shown that *daf-36* and *daf-9*mutations also enhance the *akt-1* dauer-constitutive phenotype [34]. Furthermore, *hsd-1* mutation synergizes with *akt-1* mutation to increase DAF-16/FoxO target gene expression, and DAF-16/FoxO target gene expression in *hsd-1;akt-1* double mutants requires not only DAF-16/FoxO but also DAF-12 [34]. Thus, DAF-12 may act in parallel to DAF-16/FoxO to coregulate a subset of DAF-16/FoxO target genes. *hsd-1* is expressed exclusively in the two endocrine XXX cells, where *daf-9* is also expressed [34,37,39]. Taken together, these findings suggest that DAs synthesized in the XXX cells act through DAF-12 and in parallel to AKT-1 to coregulate DAF-16/FoxO target gene expression. In contrast to *akt-1* mutation, *hsd-1* mutation does not promote nuclear translocation of a functional DAF-16::GFP fusion protein [34], indicating that HSD-1 controls DAF-16/FoxO activity without promoting DAF-16/FoxO translocation to the cytoplasm.

The role of HSD-1 in lifespan control is complex. In wild-type animals, HSD-1 does not have a major impact on adult lifespan. However, in animals with reduced IIS, HSD-1 is required for full lifespan extension [34]. This is reminiscent of the requirement for DAF-36 and DAF-9 in lifespan extension induced by germline ablation [29,40]. Surprisingly, in contrast to *daf-36* and *daf-9* mutations, which substantially shorten the lifespan of animals lacking a germline [29,40], *hsd-1* mutation does not affect lifespan extension induced by germline ablation [34]. This may be a consequence of the relative potencies of Δ^4^- and Δ^7^-DA and/or the anatomical source of distinct DAs (XXX cells *vs.* hypodermis and somatic gonad). Alternatively, HSD-1 may participate in the biosynthesis of a different steroid hormone that is required for full lifespan extension in the context of reduced IIS but dispensable for lifespan extension induced by germline ablation.

## EAK-7

In contrast to *hsd-1* and other *eak* single mutants, *eak-7* single mutants live approximately 25% longer than wild-type animals, and this lifespan extension requires DAF-16/FoxO as well as the DAF-16/FoxO cofactors HSF-1 and SMK-1 [33]. As is the case for other EAK proteins, EAK-7 inhibits nuclear DAF-16/FoxO activity without influencing its subcellular localization [33]. Notably, although *eak-7* mutation strongly enhances the dauer-constitutive phenotype of *akt-1* mutants, its enhancement of lifespan extension in *akt-1* mutants is relatively modest [33]. This is likely a consequence of the influence of the germline on DAF-16/FoxO subcellular localization; since the germline promotes the cytoplasmic sequestration of DAF-16/FoxO [24], *akt-1* mutation may not increase nuclear concentrations of DAF-16/FoxO to the extent that it does in early larval stages, before the germline has proliferated. In accordance with this, *eak-7* mutation strongly enhances lifespan extension (by ~50-80%) in animals lacking a germline [33].

**Figure 1.**
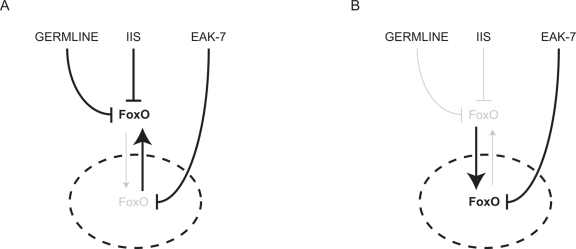
Hypothetical model of FoxO regulation by the germline, IIS, and EAK-7. The nucleus is denoted by the dashed ellipse. A. In the presence of an intact germline and IIS, most FoxO is sequestered in the cytoplasm, and EAK-7 inhibits the activity of the small fraction of cellular FoxO that resides in the nucleus. B. In animals with reduced IIS and/or germline signaling, EAK-7 has a greater influence on FoxO activity, since relative concentrations of nuclear FoxO are increased.

How EAK-7 controls nuclear DAF-16/FoxO activity is unclear. EAK-7 contains a consensus N-myristoylation (N-myr) motif and a TLDc (*T*BC- and *L*ysM-*d*omain-*c*ontaining) domain [33], the function of which is poorly understood. Interestingly, a functional EAK-7::GFP fusion protein localizes to the plasma membrane and does not appear to translocate in adult animals [33]. Thus, EAK-7 likely regulates nuclear DAF-16/FoxO activity indirectly. *eak-7* mutation increases steady-state DAF-16/FoxO protein levels in *akt-1* mutants without altering *daf-16/FoxO* transcript levels, suggesting that EAK-7 may modulate DAF-16/FoxO synthesis or turnover [33]. A hypothetical model of how EAK-7 controls DAF-16/FoxO activity in concert with IIS and the germline is shown in Figure 1.

## Potential role of the EAK pathway in diseases associated with aging

Although studies in mammalian cells have also implicated a regulatory input that controls the activity of FoxO TFs that are localized to the nucleus [41], the nature of this input is obscure. Our data suggest that the putative HSD-1 and EAK-7 mammalian orthologs Sdr42e1 and KIAA1609 may be components of this input. Recent work on mice harboring conditional knockout alleles of FoxO1 point to a potential role for FoxO TFs in the pathogenesis of Type 2 diabetes [17,18,19,20], cancer [14], and osteoporosis [16,42]; thus, it is conceivable that the EAK pathway may influence the pathogenesis of these aging-associated diseases.

The function of the putative mammalian HSD-1 ortholog Sdr42e1 is not known. Sdr42e1 mRNA is expressed in a limited number of mouse tissues, with strikingly high expression in liver [43], where FoxO1 plays an important role in metabolic homeostasis [19,20]. In contexts of insulin resistance, reduction of hepatic FoxO1 activity substantially ameliorates dysregulation of glucose metabolism in mice [19,20]. Since FoxO1 is nuclear in these circumstances [44], Sdr42e1 could play an important role in controlling hepatic FoxO1 activity and glucose homeostasis. The putative EAK-7 ortholog KIAA1609 is widely expressed in mouse tissues and is also present in mouse liver [43], indicating that KIAA1609 may also influence glucose metabolism in the context of insulin resistance. Both Sdr42e1 and KIAA1609 are expressed in mouse osteoblasts [43], where FoxO TFs promote the maintenance of normal bone mass and control metabolism nonautonomously through regulation of osteocalcin activity [16,42]; thus, EAK pathway activity in osteoblasts could influence the development of both osteoporosis and Type 2 diabetes. Intriguingly, KIAA1609 is highly expressed in mouse retinal pigment epithelium (RPE), where FoxO1 and FoxO3 are also expressed [43]. Although nothing is known about the function of FoxO TFs in RPE, the extreme levels of KIAA1609 expression in this tissue suggest that the EAK pathway could be important in regulating FoxO activity in RPE.

It is noteworthy that the genes encoding human Sdr42e1 and KIAA1609 lie within 2.5 megabases of each other at chromosome 16q23. Copy number variation in this genomic region could affect both genes coordinately, changing baseline EAK pathway activity (and, in turn, FoxO TF activity) accordingly.

## SUMMARY

The EAK pathway is a new conserved FoxO regulatory input that acts in parallel to Akt/PKB and the germline to control *C. elegans* lifespan. Continued investigation of the *C. elegans* EAK pathway promises to reveal new insights into mechanisms of FoxO regulation. In light of the potential role of FoxO TFs in mammalian aging and the pathogenesis of aging-associated diseases, EAK proteins could emerge as promising targets for the development of new drugs to treat Type 2 diabetes, cancer, and osteoporosis. Studies of Sdr42e1 and KIAA1609 function in mouse models of aging and disease will be especially revealing in this regard.
